# Supplementary material for: Characteristics of antimicrobial resistance in Escherichia coli isolated from retail meat products in North Carolina
Source: PLoS One. 2024 Jan 5;19(1):e0294099. doi: 10.1371/journal.pone.0294099 (PMC10769054; doi:10.1371/journal.pone.0294099)
Supplement: S1 Table — (DOCX) [file pone.0294099.s002.docx]

**S1 Table. Distribution of F Plasmid Replicon Sequence Types in *E. coli* isolated from retail meat products, North Carolina.**

| **Plasmid replicon sequence types** | **n = 92** | **%** |
| --- | --- | --- |
| F18:A-:B1 | 12 | 13.0 |
| F18:A-:B- | 6 | 6.5 |
| F4:A-:B20 | 6 | 6.5 |
| F18:A27:B1 | 5 | 5.4 |
| F24:A-:B1 | 4 | 4.3 |
| F18:A-:B20 | 3 | 3.3 |
| F2:A-:B- | 3 | 3.3 |
| F4:A-:B1 | 3 | 3.3 |
| F16:A-:B- | 2 | 2.2 |
| F18:A8:B- | 2 | 2.2 |
| F2:A8:B- | 2 | 2.2 |
| F34:A-:B1 | 2 | 2.2 |
| F43:A-:B- | 2 | 2.2 |
| F64:A-:B27 | 2 | 2.2 |
| F-:A8:B- | 2 | 2.2 |
| F18:A5:B1 | 2 | 2.2 |
| F-:A-:B54 | 1 | 1.1 |
| F-:A-:B77 | 1 | 1.1 |
| F-:A8:B20 | 1 | 1.1 |
| F100:A-:B- | 1 | 1.1 |
| F112:A-:B40 | 1 | 1.1 |
| F18:A-:B16 | 1 | 1.1 |
| F18:A-:B40 | 1 | 1.1 |
| F18:A-:B54 | 1 | 1.1 |
| F18:A-:B6 | 1 | 1.1 |
| F18:A-:B8 | 1 | 1.1 |
| F18:A27:B20 | 1 | 1.1 |
| F18:A6:B42 | 1 | 1.1 |
| F2:A-:B25 | 1 | 1.1 |
| F2:A6:B- | 1 | 1.1 |
| F29:A-:B- | 1 | 1.1 |
| F29:A-:B1 | 1 | 1.1 |
| F29:A8:B1 | 1 | 1.1 |
| F30:A-:B- | 1 | 1.1 |
| F33:A-:B- | 1 | 1.1 |
| F33:A19:B1 | 1 | 1.1 |
| F34:A-:B27 | 1 | 1.1 |
| F34:A27:B1 | 1 | 1.1 |
| F36:A27:B1 | 1 | 1.1 |
| F36:A5:B1 | 1 | 1.1 |
| F4:A-:B- | 1 | 1.1 |
| F40:A8:B- | 1 | 1.1 |
| F46:A-:B24 | 1 | 1.1 |
| F57:A-:B23 | 1 | 1.1 |
| F57:A8:B23 | 1 | 1.1 |
| F64:A-:B- | 1 | 1.1 |
| F67:A6:B16 | 1 | 1.1 |
| F87:A-:B- | 1 | 1.1 |
| F89:A-:B43 | 1 | 1.1 |
| F99:A-:B45 | 1 | 1.1 |
